# The wet nurses of the Hospital Real of Santiago de Compostela between 1803 and 1808

**DOI:** 10.17533/udea.iee.v42n2e03

**Published:** 2024-07-03

**Authors:** Carla Campos Villar, Emilio Rubén Pego Pérez

**Affiliations:** 1 ,* Nurse. Servicio Gallego de Salud. Santiago de Compostela, A Coruña, España. Email: carla.campos.villar@sergas.es Universidad de Santiago de Compostela Servicio Gallego de Salud Santiago de Compostela A Coruña Spain carla.campos.villar@sergas.es; 2 Nurse, Ph.D. Professor, Facultad de Enfermería de la Universidad de Santiago de Compostela. A Coruña, España. Email: emilioruben.pego@usc.es. Corresponding author. Universidad de Santiago de Compostela Facultad de Enfermería de la Universidad de Santiago de Compostela A Coruña Spain emilioruben.pego@usc.es

**Keywords:** maternal nutrition, breast feeding, infant mortality, child rearing, hospitals, smallpox vaccine, expeditions, child, orphaned, nutrición materna, lactancia materna, mortalidad infantil, crianza del niño, hospitales, vacuna contra viruela, expediciones, niños huérfanos., nutrição materna, aleitamento materno, mortalidade infantil, educação infantil, hospitais: vacina antivariólica, expedições, crianças órfãs.

## Abstract

**Objective.:**

To analyze the duties of wet nurses at the Hospital Real in Santiago de Compostela (Spain). The secondary objectives were to compare the mortality rate and distribution by parish of the foundlings under the care of the Royal House between 1803 and 1808; and to determine the origin of the Galician foundlings who participated in the Royal Philanthropic Expedition of the Smallpox Vaccine in 1803.

**Methods.:**

Historiographic study that analyzed sorted and not sorted in series indirect positional and quantitative historical sources.

**Results.:**

The duties of wet nurses during the studied period were to provide basic care and cultural instruction. The mortality rate of foundlings fluctuated during that period and their distribution by parish (functional unit of healthcare services at that time) was similar in those years, with a predominance in the provinces of A Coruña and Pontevedra. A total of 5 Galician foundlings from the House analyzed were part of the smallpox vaccine expedition, their names were Juan Antonio, Jacinto, Gerónimo María, Francisco Florencio and Juan Francisco.

**Conclusion.:**

During the observed period the wet nurses of the Hospital Real of Santiago de Compostela were in charge of pediatric care. Wet nurses were vital in the role of keeping the foundlings alive and can be considered as one of the forerunners of the pediatric nurse profession at that time.

## Resumen

**Objetivo.:**

analizar las funciones que realizaban las amas de leche en el Hospital Real de Santiago de Compostela (España). Los objetivos secundarios han sido: comparar la mortalidad y distribución por parroquias de los niños expósitos a cargo de la Real Casa entre 1803 y 1808, determinar la procedencia de los niños expósitos gallegos que participaron en la Real Expedición Filantrópica de la Vacuna de la viruela en 1803.

**Métodos.:**

Estudio historiográfico que analizó fuentes históricas posicionales indirectas y cuantitativas seriadas y no seriadas.

**Resultados.:**

las funciones de las nodrizas durante el período de estudio eran proporcionar los cuidados básicos e instrucción cultural; la mortalidad de los expósitos sufrió oscilaciones a lo largo del período analizado. La distribución por parroquias (unidad funcional de las áreas de salud en la época) fue similar en estos años, con predominancia de las provincias de A Coruña y Pontevedra. Un total de 5 niños expósitos gallegos de la Casa analizada han participado en la expedición filantrópica de la viruela, sus nombres fueron: Juan Antonio, Jacinto, Gerónimo María, Francisco Florencio y Juan Francisco.

**Conclusión.:**

Durante el período de observación, las amas de leche del Hospital Real de Santiago de Compostela realizaban cuidados pediátricos. Las nodrizas cumplieron un rol fundamental para el mantenimiento con vida de los niños expósitos y pueden ser consideradas como una de las figuras precursoras de la profesión enfermera pediátrica en la época.

## Resumo

**Objetivo.:**

Analisar as funções desempenhadas pelas nutrizes no Hospital Real de Santiago de Compostela (Espanha). Os objetivos secundários foram: comparar a mortalidade e distribuição por freguesia dos enjeitados responsáveis ​​pela Casa Real entre 1803 e 1808, determinar a origem dos enjeitados galegos que participaram na Real Expedição Filantrópica da Vacina contra a Varíola em 1803.

**Métodos.:**

Estudo historiográfico que analisou fontes históricas posicionais indiretas e quantitativas seriadas e não seriadas.

**Resultados.:**

As funções das amas de leite durante o período do estudo eram fornecer cuidados básicos e instrução cultural; A mortalidade dos enjeitados oscilou ao longo do período analisado. A distribuição por freguesias (unidade funcional das áreas de saúde da época) foi semelhante nestes anos, com predominância das províncias da Corunha e Pontevedra. Na expedição filantrópica contra a varíola participaram um total de 5 crianças galegas da Casa analisada, os seus nomes eram: Juan Antônio, Jacinto, Gerônimo María, Francisco Florencio e Juan Francisco.

**Conclusão.:**

Durante o período de observação, as nutrizes do Hospital Real de Santiago de Compostela prestaram cuidados pediátricos. As amas de leite desempenharam um papel fundamental na manutenção da vida dos enjeitados e podem ser consideradas uma das figuras precursoras da profissão de enfermagem pediátrica da época.

## Introduction

Nursing has experienced numerous changes and regulations until it became the profession it is today. In this sense, several occupations have been decisive in its shaping. Among these, it is worth highlighting wet nurses or nutrix, also known as *nodrizas,* nourice, “nourrice” or “nutrice”, a term which comes from the Latin *nutricia* (payments and salaries given to wet nurses for their work and occupation), which could be defined as women who breastfed an infant that was not their biological child.^1^

The figure of wet nurses appeared for the first time in the Classical Period, although already during the Middle Ages there had been widespread use of wet nurses to raise the successors of royalty. In order to be hired, they had to meet certain requirements: be young, married, have given birth to two or more children and pass a medical examination to evaluate breasts (appearance, quantity of milk, and shape of the nipples) and to check for venereal diseases. The employing family requested the corresponding certificate of morality issued by the parish priest of the village, who was in charge of monitoring the care of the minor, issuing to the reference foundling hospital, if necessary, a certificate confirming the death or illness of the child.^2^ The Renaissance stood out for being the historical moment when the greatest number of hired wet nurses was reached, “professionalizing” this service.^1^

In the beginning, the care of foundlings was carried out within the charitable hospitals in a specific room used for that purpose. The hospitals founded during this period included the Hospital de Santa Cruz of Barcelona (1450), the Hospital Real of Santiago de Compostela (1509), the Hospital San Juan de Dios of Granada (1553), and the Inclusa of Madrid (1572). It would not be until 1750 when the Hospitals of Foundlings were created, considered as those destined for the care of newborns who were abandoned, exposed, or entrusted to a charitable establishment. There, the personnel dedicated to breastfeeding and caring for the newborns were selected.^1^

Sometimes the parents, when leaving their child at the foundling home, left a note with the name of the baby and even the surname they wished the child to keep. This happened in the case of those citizens who, due to economic reasons, could not take care of their children but had the hope of being able to reclaim them in the near future. In the case of not having a surname, the following were given to the children: *de Inclusa* (of Foundling Home), *de todos los santos* (of All Saints), *expósito* (Foundling), among others.^3^^,^^4^

After the French Revolution in 1789, the Contemporary Era began, in which women entered the labor market, forcing many mothers to leave their children in the care of a hired wet nurse, or to abandon them in an orphanage.^4^In the second half of the eighteenth century and at the beginning of the nineteenth century, the infant mortality rate was high, and the cause of disease was linked to scientific-theoretical reasoning, both from a physical-mechanical and a social perspective. The discovery of microorganisms and vaccines allowed life expectancy to increase and the prevention of infant mortality began to gain importance.^4^

Thus, foundling homes hired wet nurses from different population centers to take care of part of the foundlings, since the sanitary conditions in the cities were not adequate due to the great demographic growth they had in such a short period of time. In addition, the institutions intended for the care of minors, due to the great demand and the few available resources, started using artificial breastfeeding, which was of animal origin.^5^

**Figure 1:**
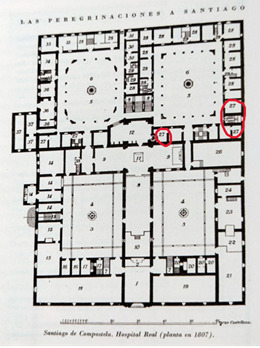
Map of the Hospital Real in 1807. The space that held the foundling home corresponds to number 27 in the image.^7^

**Figure 2:**
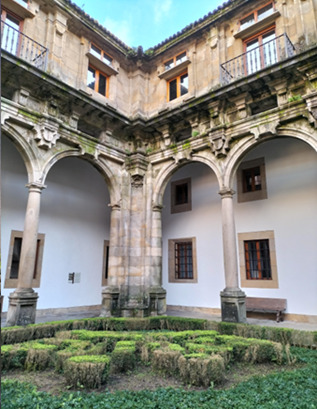
Corner of San Mateo’s Courtyard where the old foundling home is located. Original image.

The Hospital Real of Santiago de Compostela was inaugurated by the Catholic Monarchs in 1509 with the purpose of receiving the sick and pilgrims. The establishment of the foundling home occurred in the same year, but it was in the eighteenth century when it became vitally important, as the number of children in the care of this Royal House tripled. In its beginnings, the home only had one older mistress. Later, in 1736, two other wet nurses were hired (Figure 1 and 2).^6^^,^^7^

To cover the great demand, the institution hired external wet nurses, who provided care in their own homes, mostly in rural areas. From 1833 onwards, the number of foundlings decreased, so the hospital’s activity was almost entirely devoted to the care of the sick.^6^

At the beginning of the nineteenth century different campaigns were started, promoted by doctors and philosophers, aimed at raising public awareness of the need to provide good hygiene and care for infants, calling on mothers and targeting wet nurses, thus causing the employment of the latter to fall.^2^An example of an awareness campaign was the publication of Rousseau’s *Emile, or On Education*, where the figure of wet nurses was denounced as “bad mothers”, since it was claimed that charging for carrying out the activities of motherhood was immoral.^8^ Concepción Arenal, in her book *La beneficencia, la filantropía y la caridad* (Welfare, philanthropy and charity), talked about the situation of foundling homes in those times. She denounced that foundlings were overcrowded in those institutions and that wet nurses often had several children in their care, thus making their upbringing difficult.^9^

At the end of the nineteenth century, the parish priest Theodor Fliedner and his wife Friederike Münster founded the Kaiserswerth Deaconess Institute, which became the first body to define the duties of nurses in Europe. It was there that Florence Nightingale was trained, who started the first nursing school.^7^

In Spain, in 1915, nursing was professionalized through the Royal Order of May 7, 1915, which established a program of studies to obtain the official degree. Nurses had access to general, practical and specific education. Among the institutions that offered it, the following stood out: the Santa Madrona School in Barcelona inaugurated in 1917, which prepared custodial and hospital nurses; the San José and Santa Adela Schools in Madrid, which opened in 1918 and were linked to the education of Red Cross nurses; the Nursing School for Auxiliary Medical Nurses of the Mancomunidad de Catalunya, in operation since 1919; the National School of Puericulture inaugurated in 1923, specialized in assistance to the infant population; and, finally, the Nursing School of the Casa Salud de Valdecilla, founded in 1929.^10^However, Spain did not have any school that taught the specialties of visiting nurse or psychiatric nurse, among others, therefore, those nurses who wanted to specialize in these subjects had to emigrate to other European countries.^1^

For the aforementioned reasons, the general objective of this study is to determine the duties of wet nurses in the Hospital Real de Santiago de Compostela between 1803 and 1808. As secondary objectives, we aim to compare the mortality rate of foundlings under the care of the Royal House between 1803 and 1808, to compare by parish the distribution of the foundlings under the care of the Royal House between 1803 and 1808, to evaluate the delivery of foundlings to the Hospital Real of Santiago de Compostela and to determine the origin of the Galician foundlings who participated in the Royal Philanthropic Expedition of the Smallpox Vaccine of 1803.

## Methods

This study is framed within the history of nursing. To carry out this project, family and religion were selected as functional units and wet nurses as a functional element, within the functional framework of the Hospital Real of Santiago de Compostela, located in the Autonomous Community of Galicia, Spain. Initially, a preliminary search was done in the most important digital search engines to obtain the available information on the topic. Information was also collected in the Biblioteca Dixital de Galicia (Galician Digital Library) to find press releases using the term “wet nurse”. After doing so, a review of historical sources was carried out, which included a selection of books on the Hospital Real.

As an indirect positional historical source, the current Hostal dos Reis Católicos (former Hospital Real) was visited in order to obtain information about the spaces where the care of the foundlings took place. In relation to both sorted and not sorted in series quantitative historical sources, various documents from the University Historical Archive of Santiago de Compostela were consulted, both in person and through the Minerva website. The terms “Hospital Real”, “foundlings”, and “wet nurse” were chosen as search terms.

The inclusion criteria were as follows: documents that analyzed the duties of wet nurses; constitutions of the Hospital Real; registry books of payments between 1803 and 1808; registry books of entry of the foundlings in Galicia between 1803 and 1808; documents that analyzed the mortality of foundlings in Galicia between 1803 and 1808; and books of accounts and reason of expenses of the foundling home between 1803 and 1808.

The exclusion criteria were as follows: certificates, provisions, letters, declarations, capitulations, licenses, petitions, interlocutories, reports; documents of the Brotherhood of the Apostle Santiago, registers of deeds; wills; town council books; books of the sick; lawsuits; land surveys; records, powers of attorney, leases; petitions; correspondence; general documents, notes, and prescriptions.

## Results

### Results of the search strategy

The local publications of Santiago de Compostela have been filtered from the website of the Biblioteca Dixital de Galicia, thus obtaining 1048 results. None of the press releases is within the time frame studied, but, because it is of interest for the purpose of this work, we have selected the advertisement published in the *Diario de Santiago* number 23, on November 3, 1848: “Wet nurse. Ramona de Vilas, neighbor of Sta. María de Gastrar, has milk of a month and a half, more information in the Fuente Sequelo, house of the notary Don Manuel Pardo” (*Nodriza. Ramona de Vilas, vecina de Sta. María de Gastrar tiene leche de mes y medio, darán razón en la Fuente Sequelo, casa del escribano Don Manuel Pardo*).^11^

In the Minerva website 207 results were obtained, among which those corresponding to the period between 1803 and 1808 were selected, thus obtaining 14 documents. After the exclusion criteria were applied, 9 documents were finally obtained: two corresponding to books of accounts of the foundling home expenses, one book on the payment of wet nurses, five books on the distribution of foundlings by parish, and one document on the constitutions for the regulation and government of the Hospital Real. On the other hand, a publication of interest for the project has been selected from outside the established time frame due to its importance for the development of the work.

The actual inventory of the Hospital Real was searched. Applying the exclusion criteria the following documents have been discarded: 75 documents related to the registry of deeds; 290 documents related to the Brotherhood of the Apostle Santiago; 43 wills; 63 council books; 602 books of the sick; 1156 documents of lawsuits; 710 land surveys; 2486 records, powers and leases; 76 petitions; 823 correspondences; 143 documents, notes and prescriptions; and finally 941 documents related to certificates, letters, capitulations, declarations, licenses, petitions and other documents specified in the exclusion criteria. In the case of foundling documents, 483 resources were available, of which 474 were excluded because they did not correspond to the period 1803-1808, and 10 documents were finally selected. Of these files, only one of them is exclusively in paper format (*Cuadernillos de paga de amas de cría* [Wet nurses’ booklets of pay]), the other 9 are the same as those obtained from the search in Minerva.

After analyzing the sorted and not sorted in series quantitative historical sources, the following results were obtained, which are summarized in Table 1.

**Table 1:** Summary table of the documents obtained from sorted and not sorted in series quantitative historical sources.

Document name	Year	Status and characteristics	Content
Quantitative historical sources sorted in series			
*Hospital Real Expósitos,125. Libro de contas de gastos da Inclusa* (Hospital Real, Foundlings,125. Book of accounts of the foundling home).	1807	Handwritten book on parchment with liquid ink. Number of pages: 37	The document contains information on: Bread portions: 6 portions per wet nurse, of two pounds a day. Monthly expenditure averages 372 reales. Wine servings: 6 servings per wet nurse, of half a cuartillo per day. Monthly expenditure averages 90 reales. Workers’ salaries: a total of 183,532 reales. Others: oil, candles, and tallow expenditures Total, foundling home expenditures: 189,251 reales.
*Hospital Real Expósitos,126. Libro de contas de gastos da Inclusa* (Hospital Real, Foundlings,126. Book of accounts of the foundling home).	1808	Handwritten book on parchment with liquid ink. Number of pages: 37	The document contains information on: Bread portions: 6 portions per wet nurse, of two pounds a day. Monthly expenditure averages 372 reales. Wine servings: 6 servings per wet nurse, of half a cuartillo per day. Monthly expenditure averages 90 reales. Workers’ salaries: a total of 183,532 reales. Others: oil, candles, and tallow expenditures Total, foundling home expenditures: 195,251 reales.
*Hospital Real Expósitos,121. Libro de rexistro de entrega de nenos/as ás amas de cría* (Hospital Real, Foundlings,121. Registry book of foundlings’ delivery to the wet nurses).	1803-1804	Handwritten book on parchment with liquid ink. Number of pages: 498	Each of the documents contains information about the parish to which the wet nurse belonged, her name and the name of her husband, as well as the names of the children who died or were in her care.
*Hospital Real Expósitos,122. Libro de rexistro de entrega de nenos/as ás amas de cría* (Hospital Real, Foundlings,122. Registry book of foundlings’ delivery to the wet nurses).	1804-1805	Handwritten book on parchment with liquid ink. Number of pages: 397	Each of the documents contains information about the parish to which the wet nurse belonged, her name and the name of her husband, as well as the names of the children who died or were in her care.
*Hospital Real Expósitos,123. Libro de rexistro de entrega de nenos/as ás amas de cría* (Hospital Real, Foundlings,123. Registry book of foundlings’ delivery to the wet nurses).	1805-1806	Handwritten book on parchment with liquid ink. Number of pages: 470	Each of the documents contains information about the parish to which the wet nurse belonged, her name and the name of her husband, as well as the names of the children who died or were in her care.
*Hospital Real Expósitos,124. Libro de rexistro de entrega de nenos/as ás amas de cría* (Hospital Real, Foundlings,124. Registry book of foundlings’ delivery to the wet nurses).	1806-1807	Handwritten book on parchment with liquid ink. Number of pages: 510	Each of the documents contains information about the parish to which the wet nurse belonged, her name and the name of her husband, as well as the names of the children who died or were in her care.
*Hospital Real Expósitos,127. Libro de rexistro de entrega de nenos/as ás amas de cría* (Hospital Real, Foundlings,127. Registry book of foundlings’ delivery to the wet nurses).	1807-1808	Handwritten book on parchment with liquid ink. Very poorly preserved. Number of pages: 512	Each of the documents contains information about the parish to which the wet nurse belonged, her name and the name of her husband, as well as the names of the children who died or were in her care.
*Hospital Real Expósitos, 112. “Libro de pagas a las amas de cría”* (Hospital Real, Foundlings, 112. “Registry book of payments to the wet nurses”).	1796-1808	Handwritten book on parchment with liquid ink. Number of pages: 223-653	The book contains information on the salaries received by the wet nurses who took care of second-class foundlings, that is, those children older than 36 months (3 years old). From 1803 to 1805 the following annual payments are maintained: in the first year 63 reales were paid, in the second 57 reales and in the third 33 reales. From 1805 onwards, the first payment was increased to 89 reales, the second to 80 reales and the third to 91 reales. From 1807 onwards the third payment was 96 reales. There was a year in 1807-1808 in which a single payment of 179 reales was made. The salary accounts were made in July of each year. In the cases in which the foundling died, the wet nurse received the payments corresponding to the months she dedicated to the care of the child.
*Hospital Real Expósitos, 119. “Cuadernillos de paga de amas de cría de expósitos de 1ª clase”* (Hospital Real, Foundlings, 112. “Booklets of pay of first-class foundlings’ wet nurses”).	1802-1808	Several booklets held together by thread and a cover with the title: “*Cuaderno de paga de expósitos de primera clase, desde 1802 hasta 1808* (First-class foundlings’ pay notebook from 1802 to 1808).” Each document is signed by the authorities of the Royal House of the time, among them, Antonia de la Concha (main mistress). It contains an index of parishes.	1801: there was a misplaced document from this year which reflects the total expenditure on the salaries of the wet nurses: 56,036 reales. 1802: minimum wage 33, maximum 171. Mode: 85 reales. Total spent: 50,717 reales. 1803: minimum wage 28, maximum 175. Mode: 85 reales. Total spent: 45,978 reales. 1804: minimum wage 35, maximum 152. Mode: 86 reales. Total spent: 54,609 reales. 1805: minimum wage 20, maximum 97. Mode: 85 reales. Total spent: 34,934 reales. 1806: minimum wage 31, maximum 164. Mode: 121 reales. Total spent: 58,151 reales. 1807: minimum wage 37, maximum 372. Mode: 251 reales. Total spent: 137,284 reales. 1808: minimum wage 15, maximum 340. Mode: 238 reales. Total spent: 153,203 reales.
Quantitative historical sources not sorted in series			
*Constituciones para el régimen y gobierno del Hospital Real de la ciudad de Santiago y administración, cuenta y razón de sus bienes y rentas* (Constitutions for the regulation and government of the Hospital Real of the city of Santiago and its management, account and reason of its goods and revenues).	1804	Set of typewritten documents on white paper. Numbered page by page. With the seal of the Hospital Real. Number of pages: 236	The document provided information on how the Hospital Real was to be organized and managed. It specified the ideal number of each type of worker, how the hierarchy was to be established, and how the Boards were to be appointed, among other matters. Regarding the care of foundlings, it mandated that baptism was to be given immediately once the child had entered the foundling home.
*Cartilla o método que se observará en la Inclusa del gran Hospital Nacional de Santiago para con sus expósitos. Dispuesta por la Junta Interina del mismo* (Booklet or method to be observed in the foundling home of the great National Hospital of Santiago for its foundlings. Disposed by the Acting Board of the hospital).	1821	Set of typewritten documents on white paper. Numbered page by page. With the seal of the Hospital Real. Number of pages: 16	The document provided information on how the wet nurses and nursemaids were to carry out their work (feeding, providing warmth, and cleaning the infants). It also established the distribution of the foundlings and how the rooms that housed them were to be set up.

Regarding the information obtained from the indirect positional historical source, eight experts in the field were contacted, one of whom did not reply, two of them recommended reading articles that have not been included in the paper because they do not study the historical period in question, two recommended contacting other experts in the field, and two other experts recommended reading the book “*El hospital Real de Santiago de Compostela y la hospitalidad en el camino de peregrinación*” (The Hospital Real of Santiago de Compostela and hospitality on the pilgrimage way) and the congress lecture “Amas, enfermeras, mozas y lavanderas. Los niveles de vida de las trabajadoras del Hospital Real de Santiago (1800-1930) (Mistresses, nurses, maids and laundresses. The living standards of the female workers at the Hospital Real of Santiago [1800-1930])”, both of which have been included as bibliographic references for the writing of this paper. Finally, Isidoro Rodríguez Pérez, professor of Nursing History at the University of Santiago de Compostela, at the Lugo campus, agreed to do a complete personal interview in which he emphasizes that at that time there were foundling homes in the cities of the Autonomous Community of Galicia, where children up to four to five years old were sent to the care of a paid wet nurse, which coincided with the regency of Isabel Zendal of the foundling home in A Coruña. Lastly, he emphasizes that, as the literature reports, infant mortality in general was high, although it is very difficult to determine the causes associated with malpractice or with the diseases that affected that era.

## Results

For the main objective, “to determine the duties of wet nurses in the Hospital Real de Santiago de Compostela between 1803 and 1808”, it has been analyzed that the main task of the wet nurse was to keep alive the foundling or foundlings in her charge. To this end, she breastfed the child, provided basic care (hygiene, sleep, etc.), and in most cases was also responsible for the subsequent education, since the surviving children were often fostered by their wet nurses. Therefore, these women were not only in charge of perpetuating the life of the child, but also of the social and cultural aspects of the child’s life.^12^^,^^13^^)^

The duties of the wet nurses in the hospital were classified into four: warmth, cleanliness, food, and sleep.^12^ They had to look after the children and this included watching over the turnstile located in one of the hospital’s windows where the parents left the children. Therefore, they were the infant’s first contact with the institution.^4^^,^^14^ The turnstile had to have bells that alerted the wet nurse on duty of the arrival of a new foundling. At this first moment, they had to caress and undress him, note the condition in which the child arrived and if they brought with them a baptismal certificate. In the case of children who were in a very serious condition, they were to notify the manager so that baptism could be performed.^12^^,^^14^

The warmth had to be maintained, preferably with a brazier in each of the rooms that housed the children; there could be neither fumes nor toxins. To provide warmth to the newcomer, a bath in warm water was given and the infant was placed in a cradle with clean clothes.^4^^,^^12^As for the food, it depended on the needs and age of each foundling; for those who were very critically or seriously ill, a wet nurse was assigned to them; on the other hand, for those who were robust, cow’s or goat’s milk was prescribed, which was watered down if required by the doctor. From seven months of age onwards, the nurses were required to prepare more substantial porridges such as rice soups, bread soaked in milk or barley creams with egg yolk. From the age of one year or fifteen months, depending on the child’s needs, whole foods were introduced.^12^

For better care, the foundlings were classified into three groups: healthy, sick, and suspicious. The foundling nursemaid was in charge of the healthy ones (a widow who did the same job as the wet nurses, but administered artificial lactation with feeding bottles, which were individual and had to be washed after each use), and the suspicious and sick ones were assigned a wet nurse or a nursemaid depending on the needs of each child. The rooms where care was provided were white, clean, and warm. Each of the cradles was numbered and had a board on which the feedings that had been provided were noted. For every 6 cradles there was a wet nurse or a nursemaid who looked after them.^12^

On the other hand, wet nurses who took care of the children in their own homes were in charge of transporting them from the capital to the town where they were going to live with the child, which was arduous, since they did not have the means nor adequate roads to do so. These women provided not only the aforementioned care, but also gave the child the opportunity to live in a family, since they lived together with their husbands and children.^6^

For the secondary objective “To compare the mortality rate of foundlings in the care of the Royal House between 1803 and 1808”, this study presents the data in Table 2. The data shows that the mortality rate of foundlings varies according to the period, with the lowest in 1806-1807 and the highest in 1807-1808. As for those who died before registering, they represent between 9% and 19.23% of the total mortality rate.^15^^-^^20^

**Table 2:** Royal House’s foundlings’ mortality rate between 1803-1808

Year	Total Foundlings	No. of Dead Foundlings	No. of Live Foundlings
May 1803 - April 1804	545	351 (64.4%) Deceased before being registered: 54 (10%)	194 (33.6%)
May 1804 - April 1805	514	286 (55.7%) Deceased before being registered: 55 (19.23%) Deceased before leaving the foundling home: 197	228 (44.3%)
May 1805 - April 1806	606	383 (63.2%) Deceased before being registered: 64 (17%) Deceased before leaving the foundling home: 202	223 (36.8%)
May 1806 - April 1807	670	362 (54%) Deceased before being registered: 33 (9%) Deceased before leaving the foundling home: 174	308 (46%)
May 1807 - April 1808	640	477 (74%) Deceased before being registered: 43 (9%) Deceased before leaving the foundling home: 196	163 (26%)

For the secondary objective “To compare the distribution by parish of the foundlings under the care of the Royal House between 1803 and 1808”.^15^^-^^19^ Next, the number of parishes included in the distribution and the point farthest from Santiago de Compostela, the main seat of the Royal House, is detailed by year. In 1803-1804 there were a total of 192 parishes and the farthest point corresponds to Burela, at a distance of 160 km; in 1804-1805 there were a total of 251 parishes and Viana do Bolo was the farthest point, at a distance of 220 km; in 1805-1806 there were a total of 236 parishes and the farthest point on the map corresponds to Viveiro (140 km); in 1806-1807 there were a total of 246 parishes and the farthest point was Foz (153 km) and finally in 1807-1808 there were 221 parishes and the farthest point was Viveiro (140 km).^15^^-^^20^

**Figure 3:**
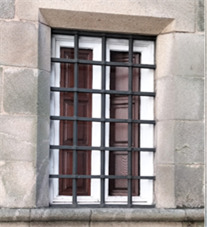
View of the window from San Francisco Street

For the secondary objective, “To evaluate the delivery of foundlings in the Hospital Real of Santiago de Compostela”, the following information was found during the visit to the current *Hostal*, in the plaque number 30 placed by the *Hostal*’s Museum. The plaque describes how the exchange of children was carried out: “In San Francisco Street there was a window with a bell and a turnstile like the one in cloistered convents. Someone would knock, wait until they heard ‘*Ave Maria Purisima* (Holy Mother of God)’ and then deposit the newborn.” The window in which the infants were placed has been photographed. Nowadays it is closed with bars and no longer has the turnstile that it had in former times (Figure 3).

Finally, for the secondary objective “To determine the origin of the Galician foundlings who participated in the Royal Philanthropic Expedition of the Smallpox Vaccine of 1803”, the expedition was carried out in 1803 and promoted by several high officials of the Spanish healthcare and government of the time. It is worth mentioning the participation of Isabel Zendal, nurse and head of the foundling home of A Coruña, who was in charge of the care of the 21 foundlings who were inoculated with the smallpox vaccine across the Atlantic to Mexico.^21^^,^^22^

**Figure 4:**
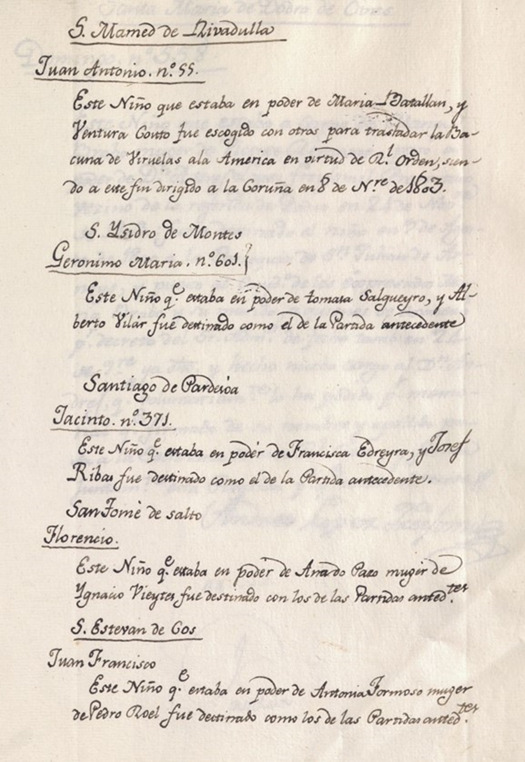
Document extracted from the Foundling Hospital Real (1803-1804). Information corresponding to the children who were selected to carry the vaccine to America in the Royal Philanthropic Expedition of the Smallpox Vaccine ^15^

Five of the carrier children belonged to the Royal House, among them, Juan Antonio, whose wet nurse was named María Batallan and her husband Ventura de Couto, from the parish of San Mamede de Rivadulla; Jacinto, who was under the care of Francisca Edreira and Josef Rivas from the parish of Santiago de Pardesoa; Gerónimo María, whose wet nurse was Tomasa Salgueiro and her husband Alberto Vilar, coming from San Isidro de Montes; Francisco Florencio under the care of Ignacio Vieites and Ana de Pazo, neighbors of San Tomé de Salto; and, finally, Juan Francisco, under the care of Antonia Formoso and Pedro Roel, from the parish of San Estevan de Cos (Figure 4).^15^^,^^21^^,^^22^

**Figure 5:**
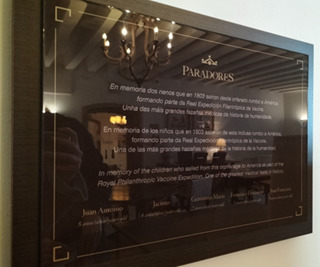
Plaque commemorating the children who participated in the Royal Philanthropic Vaccine Expedition

Also, during the visit to the *Hostal*’s Museum, a plaque commemorating the five children who participated in the Royal Expedition was found to the left of the window where the foundlings were deposited (Figure 5).

## Discussion

The importance of this work lies in viewing history as a tool for understanding the origins of the nursing profession, since it is the first step in recognizing and studying nursing as we know it today.^1^Josep Fontana, in the prologue of the book *Los métodos de la Historia* (The Methods of History), written by Cardoso and Pérez Brignoli, emphasizes the importance of the knowledge of history in order to understand the present and future; “It would be good to begin to teach history as a system of research: as a set of methods whose main purpose is to help people to understand, through the deciphering of their past, the reasons that explain their present situation and the perspectives from which they must start in the elaboration of their future”.^23^

The history of nursing is as old as humanity. Since ancient times there were one or several figures in charge of providing care to the population. Each of them played a specific role, so it is necessary to give each part of the system the importance it deserves.^1^

This paper focuses on the years 1803 to 1808. It was decided to study these years because it was a difficult time for this profession, as it was in this historical moment when the greatest boom in the hiring of wet nurses was reached and when, later, their work was questioned. Therefore, the purpose of this study is to have a broader vision of the pediatric care performed by wet nurses as one of the precursor figures of the pediatric nursing profession at the time.^1^^,^^2^^,^^5^

The duties performed by wet nurses at the Hospital Real of Santiago de Compostela are very similar to those carried out by wet nurses in different parts of the world. In the case of Portugal, in Oporto, there were different types of wet nurses; the *amas de dentro*: wet nurses who were inside the foundling home, who watched over the turnstile; the *amas de empréstito*, who were hired when there was an excess of foundlings; and, finally, the *amas de fora*: those who raised children in their homes until they were 7 years old. In England, in London, wet nurses were women without resources who carried out this work due to the good salaries they received. They raised between two and three children. France already had in 1769 a specific organization for the hiring of wet nurses and, in the case of Brazil, the wet nurse was a slave of the European descendants, she was separated from her biological child at birth and was forced to breastfeed her master’s child.

Along with hygiene, warmth and food, a social relationship was created not only between the wet nurse and the foundling, but also between the infant and the nurse's children. This bond was called milk kinship, which refers to the “adoption” of the foundling by the family, being included in the family dynamics as a child.^2^In all countries the same dynamic occurred; the wet nurse was a woman without resources or education, who performed this work in order to earn a living. She had to be under the protection of a man (husband, father and/or parish priest) and needed his permission to be able to perform this work.^2^

The average infant mortality of the foundlings from 1803 to 1808 extracted from the documents studied was 62.24%. In Galicia, between 1750 and 1810, the average infant mortality was 46.8%. It should be noted that this percentage includes the entire infant population at the time, whether they were foundlings or not.^23^ Therefore, when comparing these percentages, all the variables that may be involved in the difference in mortality should be taken into account, such as lack of food, greater contact with other infants (which in turn implies a greater risk of contagion of exanthematous diseases), and the transfer from the foundling home to the parishes, among others.^4^^,^^24^ On the other hand, when comparing this percentage with the mortality rate for the year 1829, the one for that year was 49.7%;^25^ thus, it can be observed that after the publication of the document “Booklet or method to be observed in the foundling home of the great National Hospital of Santiago for its foundlings”^12^the situation in the Royal House improved.

The distribution of foundlings by parish between 1803 and 1808 was similar, with a greater tendency towards the areas closest to Santiago de Compostela, being present in the four provinces, but to a greater extent in A Coruña and Pontevedra. If we compare it with the distribution established for 1829, ^25^ it continues to be similar to that obtained at the beginning of the century.

The distribution of foundlings by parish in 1750. In the accompanying text, it is specified that the maximum distance between the parishes receiving foundlings and the Royal House was approximately 8 leagues (38 kilometers).^6^

The deposit of foundlings through the turnstile was a widespread practice in the foundling homes, which allowed the child to be deposited without having to be seen. With this, the hospices themselves wanted to protect the minor, since, before the installation of the turnstile, they were often abandoned at the doors of convents, churches and foundling homes, causing the death of many of them by hypothermia during the night.^4^^,^^24^Two of the children destined for the Royal Smallpox Vaccine Expedition belonged to the province of Pontevedra and the other three to the province of A Coruña, in accordance with the dynamics of distribution corresponding to the year 1803. The participation of children in labor was a widespread practice among the society of the time; both families and institutions used them as labor for the execution of various tasks. Among other purposes, they were used as labor for medical or scientific purposes. This gave rise to the so-called “vacciniferous children”, because children had not developed immunization against smallpox and this guaranteed the expedition’s success.^21^

The publication of advertisements for wet nurses in the local press was not an isolated event, as many women opted for this method of obtaining work. In this way it was wet nurses themselves who offered their services. This type of advertising was mainly directed at private individuals.^2^

With regard to the salaries received by wet nurses in Santiago de Compostela, in comparison with other cities at the end of the eighteenth century; the salary was 30 reales a month for four years in Cartagena, without the possibility of returning the foundling; in Barcelona, 12 pounds a year for external wet nurses during lactation and 50 reales in the following 5 years; 60 reales a month as an internal wet nurse and 30 reales for two years as an external wet nurse in the foundling home of Lorca; but in Granada it was the lowest, being 16 reales a month during lactation and 11 in the following years.^3^

If we compare the salaries received by wet nurses with those of the other workers of the Hospital Real between 1803 and 1808, they had the third best salary; only the senior nurse and Antonia de la Concha, senior mistress of the foundling home in Santiago de Compostela from 1794 to 1829, were above them.^26^^,^^27^

When analyzing the expenses of the foundling home in 1807 and 1808, the cost of bread for wet nurses was 2.35% of the total in 1807 and 2.88% in 1808, while the cost of wine was 0.57% of the total in 1807 and 0.55% of the total in 1808. If we compare it with other institutions destined to the care of foundlings, its expenses at the end of the eighteenth century were the following: 24,000 reales per year in Cartagena, 37,492 reales per year in Lorca and 370,000 reales in Madrid.^3^^,^^28^^,^^29^

So, even though the Royal House was above Lorca and Cartagena in terms of foundling home expenses, it was still the one with the worst working conditions for wet nurses, ranking as the worst paid of the most important Spanish foundling homes.^3^^,^^30^

Finally, healthcare activity in the Hospital Real ceased in 1953, when care was transferred to the new hospital in the city and the building became a national inn for lodging^6^.

The main limitations of this work lie in the fact that due to the dates of the primary sources used as bibliography for the writing of this manuscript (nineteenth century), all the documentation is handwritten in ink, implying difficulties in its reading. Additionally, some of the documents consulted were deteriorated (humidity, traces of ash after reading with candles...), so that sometimes it was impossible to read some of them completely. Regarding the secondary sources (books, newspaper articles, maps, papers) selected, they have been rewritten after the period studied, so biases could have occurred in the reediting process.

### Conclusion

The duties performed by wet nurses at the Hospital Real between 1803 and 1808 were to provide basic care (warmth, feeding, hygiene and sleep) and to educate the foundlings in cultural and social aspects. They provided care for the pediatric community, therefore, they were the predecessors in specialized neonatal care following a holistic view of it before the creation of the specialty of pediatric nursing, since at that time nurses carried out generalist care.

The mortality rate of foundlings under the care of the Royal House between 1803 and 1808 fluctuated, with the lowest in the 1806-1807 interval with a mortality rate of 54%, and the highest between 1807-1808 with a mortality rate of 74%. The distribution of the foundlings by parish under the care of the Royal House between 1803 and 1808 was very similar, with a predominance in the provinces of A Coruña and Pontevedra and very few in the provinces of Ourense and Lugo. The maximum distance reached in that period was 220 km. 

The foundlings were brought to the Hospital Real of Santiago de Compostela through a turnstile located in the first window that faced San Francisco Street. The origins of the Galician foundlings who participated in the Royal Smallpox Vaccine Expedition were as follows: Juan Antonio, from the parish of San Mamede de Rivadulla; Jacinto, from Santiago de Pardesoa; Gerónimo María, from San Isidro de Montes; Francisco Florencio, from San Tomé de Salto; and finally, Juan Francisco, from San Estevan de Cos.

The purpose of this paper is to show the fundamental work carried out by wet nurses throughout history, since they were the essential link to carry out holistic neonatal care at that time and the precursors of the pediatric nursing specialty.

## References

[1] Siles González J (2009). Historia de la enfermería.

[2] Martínez Sabater A (2014). Las nodrizas y su papel en el desarrollo de la sociedad española. Una visión transdisciplinar. Las nodrizas en la prensa española del siglo XIX y principios del siglo XX.

[3] de la Fuente Galán MP (1997). La situación de las inclusas en el siglo XVIII. La encuesta de 1790. Chronica Nova.

[4] de Pablo Gafás A (1997). Niños expósitos y medicina infantil en España a principios del siglo XIX. Revista de estudios históricos de las ciencias médicas, 3ª época.

[5] Montiel Pastor J (2005). La infancia abandonada en Cataluña 1800-1945. Un análisis cuantitativo.

[6] García Iglesias JM (2004). El Hospital Real de Santiago de Compostela y la hospitalidad en el Camino de Peregrinación.

[7] Cordeiro Rodríguez M (2018). Formación de Florence Nightingale en la Institución de las Diaconisas de Kaiserswerth: análisis de documentos originales. Revista Iberoamericana de Educación e Investigación en Enfermería.

[8] Rousseau JJ (2011). Emilio o de la educación.

[9] Arenal C (1894). La beneficencia, la filantropía y la caridad.

[10] Pego Pérez ER, Rodríguez Pérez I, Bermello López L (2022). Curso y final de la vida profesional de Elvira López Mourín. Revista Ene de Enfermería.

[11] Biblioteca Dixital de Galicia (1848). Diario de Santiago: Núm 23.

[12] Montero JF (1821). Cartilla o método que se observará en la Inclusa del gran Hospital Nacional de Santiago para con sus expósitos. Dispuesta por la Junta Interina del mismo.

[13] Rodríguez Pérez I (2023). En discusión con Carla Campos.

[14] Imprenta Real (1804). Constituciones para el régimen y gobierno del Hospital Real de la ciudad de Santiago y administración, cuenta y razón de sus bienes y rentas.

[15] Hospital Real Expósitos (1803-1804). Libro de rexistro de entrega de nenos/as ás amas de cría.

[16] Hospital Real Expósitos (1805). Libro de rexistro de entrega de nenos/as ás amas de cría.

[17] Hospital Real Expósitos (1806). Libro de rexistro de entrega de nenos/as ás amas de cría.

[18] Hospital Real Expósitos (1807). Libro de rexistro de entrega de nenos/as ás amas de cría.

[19] Hospital Real Expósitos (1808). Libro de rexistro de entrega de nenos/as ás amas de cría.

[20] Hospital Real Expósitos 195. Cuadernillos de paga de amas de cría de expósitos de primera clase.

[21] Ramírez Martín SM (2003). El niño y la vacuna de la viruela rumbo a América. Rev. Complutense de historia de América.

[22] Varela S (2022). Juan Antonio, el niño de Friol que llevó en 1803 la viruela a América para salvar miles de vidas. La voz de Galicia.

[23] Pérez Brignoli H., Cardoso C (1976). Los métodos de la historia.

[24] Martínez Rodríguez E (1992). La mortalidad infantil y juvenil en la Galicia urbana del Antiguo Régimen. Santiago de Compostela 1731-1810. OHM: Obradoiro de Historia Moderna.

[25] Hospital Real Expósitos (1829). Certificaciones de párrocos sobre defunciones y crianza de expósitos residentes en sus feligresías. Documentos históricos (Exp. 3),.

[B26] Alija Domínguez M (2022). Amas, enfermeras, mozas y lavanderas. Los niveles de vida de las trabajadoras del Hospital Real de Santiago (1800-1930).

[27] Hospital Real Expósitos (1808). Libro de pagas ás amas de cría.

[28] Hospital Real Expósitos (1807). Libro de contas de gastos da Inclusa.

[29] Hospital Real Expósitos (1808). Libro de contas de gastos da Inclusa.

[30] Sarasúa García C Publicaciones de la Universitat d´Alacant.

